# Kluai Hin (*Musa sapientum* Linn.) peel as a source of functional polyphenols identified by HPLC-ESI-QTOF-MS and its potential antidiabetic function

**DOI:** 10.1038/s41598-022-08008-3

**Published:** 2022-03-09

**Authors:** Patthamawadee Tongkaew, Anna Tohraman, Ramlatee Bungaramphai, Chalermchai Mitrpant, Ebru Aydin

**Affiliations:** 1 Department of Food Science and Nutrition, Faculty of Science and Technology, Prince of Songkla University, Pattani Campus, Pattani, 94000 Thailand; 2 Department of Biochemistry, Faculty of Medicine Siriraj Hospital, Mahidol University, Bangkok, 10700 Thailand; 3 Department of Food Engineering, Suleyman Demirel University, Isparta, 32260 Turkey

**Keywords:** Nutrition, Liquid chromatography

## Abstract

To date, information on the polyphenolic composition of Kluai Hin banana peel and pulp and the potential antidiabetic activity of its major active compounds is limited. This study aimed to identify polyphenols in extracts of fresh and freeze-dried Kluai Hin banana peel and pulp (methanol:water; M:W, 80:20 for flavonoids and acetone:water:acetic acid; A:W:A, 50:49:1 for phenolic acids) by RP-HPLC-DAD and HPLC-ESI-QTOF-MS. Additionally, inhibition of α-amylase and α-glucosidase activities was investigated with crude extracts from Kluai Hin banana peel and pulp, and compared with its major polyphenols ((+)-catechin, (−)-epicatechin and gallic acid) and the antidiabetic drug acarbose. (−)-Gallocatechin was the most abundant polyphenol and was detected in all fresh and freeze-dried pulp and peel extracts by RP-HPLC-DAD. Furthermore, unidentified polyphenol peaks of Kluai Hin were further explored by HPLC-ESI-QTOF-MS. The A:W:A fresh peel extract contained more total phenolic content (811.56 mg GAE/100 g) than the freeze-dried peel (565.03 mg GAE/100 g). A:W:A extraction of the fresh and freeze-dried peel of exhibited IC_50_ values for α-amylase activity 2.66 ± 0.07 mg/ml and 2.97 ± 0.00 mg/ml, respectively, but its inhibitory activity was lower than acarbose (IC_50_ = 0.25 ± 0.01 mg/ml). Peel extracts inhibited α-glucosidase activity, whereas pulp extracts had no effect. In addition, all standards, except gallocatechin, activated α-amylase activity, while, gallocatechin inhibited α-glucosidase activity better than acarbose. Therefore, we propose a further investigation into the use of Kluai Hin banana peel as a potential functional food for the management of postprandial glycaemic response to reduce diabetes risk and in the management of diabetes with a commercial drug.

## Introduction

Banana (*Musa spp.*) is one of the most popular fruits in the world in terms of economic and nutritional value. Banana is not only a cheap food source but also contains numerous nutrients and non-nutrients, such as polyphenols^1,2^. Bananas are found in many different types and species in Thailand. However, in a southern area of Yala, Thailand, there is another type of unique banana called "Kluai Hin". Kluai Hin is a local banana belonging to the genus *Musa* from the family *Sapientum*. Although Kluai Hin is not an economic banana but still ensure high income for farmers. This is due to its unique characteristics, such as sweet taste and chewy texture. Furthermore, Kluai Hin has a very thick peel that protects the fruit during transportation and provides a longer storage time. It may be eaten fresh, but the local people mostly boil or toast it to generate a sweeter taste. In addition, the raw fruit of Kluai Hin is used as an astringent or made into various types of processed Kluai Hin products. Based on the literature, different parts of banana was investigated and various types of polyphenols and other secondary metabolites were obtained depending on extraction solution, stage and storage, area of cultivars and analytical methods^3–8^. The estimated amount of total phenol was obtained by the Folin-Ciocalteu method, while, the reverse phase-high-performance liquid chromatography-diode array detector (RP-HPLC-DAD) is more specific for identification and quantification of polyphenols compared to standards. Furthermore, an advanced, high sensitive and effective method, liquid chromatography coupled with electrospray-ionization and quadrupole time-of-flight mass spectrometry (HPLC-ESI-QTOF-MS) was used to characterize diverse and complex polyphenols from different fruit peels, including banana peel^9^.

Several in vitro and in vivo studies have demonstrated the health-promoting effects after consumption of polyphenol-rich bananas. For example, antioxidant, anti-inflammatory and antidiabetic activities^2,10–17^. The interest in natural foods (rich in polyphenols) is increasing as an alternative therapeutic approach in the management of diabetes due to no side effects. On the other hand, the drug used to treat type 2 DM, acarbose, may lead to several complications, such as diarrhoea, bloating and upset stomach^14,18^. Therefore, researchers have started to focus on the antidiabetic activity of dietary polyphenol-rich foods, such as α-amylase and α-glucosidase inhibitory activity^8,14,19–21^. Furthermore, some studies revealed that polyphenols of some parts of bananas inhibit these two key enzymes for carbohydrate digestion^8,22^.

The information of polyphenol compositions and the biological effect of local banana, Kluai Hin is limited. Due to the thick peel of Kluai Hin, it may contain various types of polyphenols more than other banana strains. Additionally, the polyphenol content of Kluai Hin pulp or other parts may be different. Therefore, the present study aimed to investigate the Kluai Hin peel and pulp polyphenols by RP-HPLC-DAD and HPLC-ESI-QTOF-MS along with their inhibitory activity on α-amylase and α-glucosidase enzymes. The antidiabetic activity was also compared with acarbose and both peel and pulp's major polyphenol content: (−)-gallocatechin, (+)-catechin, (−)-epicatechin and gallic acid. This study hypothesised that different parts of the Kluai Hin banana (pulp and peel) will contain different types of polyphenols using different extraction solutions. In addition, its extracts and the main polyphenols explored in Kluai Hin could inhibit these digestive enzymes as a very similar action to the pharmaceutical drug, acarbose. Therefore, the Kluai Hin banana is a potential candidate for a functional source of polyphenols and possible delays carbohydrate digestion and postprandial glucose absorption through inhibiting α-amylase and α-glucosidase activity, resulting in higher consumption and decrease the risk of diabetes. Additionally, Kluai Hin peels, a natural by-product of banana, will be utilized more as a functional ingredient in functional food development.

## Materials and methods

### Sample preparation and extraction

Banana, Kluai Hin (*Musa sapientum* Linn.) at the mature stage^2^ with a yellow-green group (15B) of colour peel (RHS colour chart) was purchased from the local market in Yala, Thailand. Sample preparation was adapted from Guiné et al.^23^ with a slight modification. Fresh Kluai Hin after cleaning and peeling were cut into small pieces (1 × 1 cm). 500 g of fresh sample was extracted directly, while the freeze-dried sample was ground and filtrated using 100 mesh sieves (particle size < 150 micron) and kept in brown bottles at − 20 °C until further analysis. All samples were extracted according to the method described by Tsamo et al.^5^ with a little modification. 2.5 g of each sample fresh and freeze-dried from both pulp and peel were extracted with 25 ml of two different extraction solutions; methanol: water (80:20) for extracting flavonoids and acetone:water:acetic acid (50:49:1) for extracting phenolic acids. To encourage the homogenisation and the solubility of polyphenols in different solvent types, the sample was vortexed for 1 min. Subsequently, the sample was centrifuged at 5000*g* 4 °C for 10 min. The supernatant was collected into a new tube. The sediment was then re-extracted. All supernatants were collected and evaporated to dryness with a centrifugal evaporator at 37 °C to obtain dry samples for analysis.

### Detection of polyphenols using reverse-phase high performance liquid chromatography coupled with diode array detector (RP-HPLC-DAD)

RP-HPLC-DAD was performed according to Lai et al.^10^ with a modification of the gradient elution program. The dry sample was reconstituted with 1000 µL of 50% ethanol. Each sample was filtrated through 0.45 µm PTFE membrane filters and 10 µl was injected directly. The HPLC analysis was carried out using Agilent 1100 series-coupled to a binary pump, a diode-array detector, an autosampler and a column compartment (kept at 25 °C). Separation was performed on a C18 column (Poroshell 120 EC-C18 4.6 × 100 mm, 2.7 2.5 µm) using a diode array detector (DAD) at 280 nm for gallic, (−)-gallocatechin, (+)-catechin and (−)-epicatechin. Phenolic compounds were separated using the mobile phase A (DI water and 0.1% formic acid) and B (acetonitrile and 0.1% formic acid), elution gradients as follows: 0–10 min, 5–15% B; 10–20 min, 15% B; 20–30 min, 15–35% B; 30–35 min, 35–100% B; 35–40 min, 100% B;40–41 min, 100–5% B; 41–46 min, 5% B, at a flow rate of 0.5 ml/min. Spectral data for all peaks were accumulated in the range of 190–400 nm and compared to authentic standards of gallic, (−)-gallocatechin, (+)-catechin and (−)-epicatechin.

### HPLC-ESI-QTOF-MS analysis

The HPLC system 1290 Infinity II liquid chromatography was coupled to 6545 quadrupole-time of flight mass spectrometer (LC-QTOF-MS, Agilent Technologies, USA) via an electrospray ionization source (ESI) with Dual Agilent Jet Stream Electrospray Ionization (Dual AJS. ESI). The Zorbax Eclipse Plus Rapid Resolution High Definition (RRHD) columns C18 1.8 μm, 2.1 mm i.d., 150 mm column (Agilent Technologies, USA) and a mobile phase consisting of acetonitrile and 0.1% formic acid (phase B) and water and 0.1% formic acid (phase A). The gradient method was as follows: 0–5 min, 5–5% B; 5–10 min, 5–15% B; 10–25 min, 15–15% B; 25–30 min, 15–100%B; 30–35 min, 100–100% B; 35–40 min, 100–5% B; 40–45 min and 5–5% B. The flow rate was 0.2 ml/min at 25 °C with an injection volume of 5 μL. Mass spectra was collected with acquisition ranging from m/z 100 to m/z 1500 and ESI ionization parameters were determined as follows; sheath gas at 275 °C with a flow of 12 L/min, drying gas at 325 °C with a flow of 13 L/min, OCT 1 RF Vpp 750 V, nebulizer at 35 psi, different collision energy using N2. Automatic MS/MS experiments were carried out using collision energies (10 eV, 20 eV and 40 eV). MassHunter Workstation software (Agilent Technologies, Santa Clara, CA, USA) was used for integration and data elaboration.

Identification of polyphenols in the various extracts was identified using previously reported studies in the literature^3^ depending on the full mass and unique mass fragmentation spectrum (METLIN and several online databases such as Phenol-Explorer (www.phenol-explorer.eu), SciFinder-Scholar (https://scifinder.cas.org), MassBank (http://www.massbank.jp/), ChemSpider (http://www.chemspider.com), and PubChem (https://pubchem.ncbi.nlm.nih.gov)).

### Determination of total phenol content

The total phenol content (TPC) was determined according to Sulaiman et al.^24^. Each extracts (500 µl, 1 mg/ml) was oxidized using 1.0 ml of 10% Folin–Ciocalteau reagent (v/v). After that, the reaction mixture was neutralized by 3.0 ml of 7.5% sodium carbonate. The reaction mixture was then incubated at room temperature for 2 h in the dark. Finally, the absorbance was measured at 760 nm using a spectrophotometer and TPC was calculated and expressed as gallic acid equivalent (GAE).

### α-Amylase inhibition assay

The α-amylase activity was performed with a slight modification according to two different studies(Oboh et al.^14^ Tan et al.^25^). To study the inhibitory activity of banana extracts, 250 µl of each sample (0–10 mg/ml of banana extracts or a positive control, acarbose) were mixed with porcine pancreatic α-amylase (EC 3.2.1.1; 0.5 mg/ml) in 0.02 M sodium phosphate buffer (pH 6.9 with 0.006 M NaCl at 250 µl volume). For the analysis of standard polyphenols, 250 µl of each sample (0–350 µg/ml of standard polyphenols or acarbose) was mixed with 250 µl of porcine pancreatic α-amylase. Consequently, all of the samples were incubated at 37 °C for 10 min. Then, 250 µl of 1% starch solution in the same solution buffer was added to the reaction mixture. Thereafter, the reaction mixture was incubated at 37 °C for 10 min. The reaction was stopped by adding 250 µl of 3,5-dinitrosalicylic acid (DNS) reagent, incubated in a boiling water bath for 5 min, and cooled to room temperature. 10 ml of distilled water was added to reaction mixture and measured at the wavelength of 540 nm using a spectrophotometer. The α-amylase inhibitory activity was expressed as inhibition (%).

$${\text{Inhibition}}\,{\text{(\% )}} = \left\lfloor {{1} - (({\text{Asample}} - {\text{Acontrol}})/({\text{A}}\_{\text{test}} - {\text{A}}\_{\text{blank}}))} \right\rfloor \times {1}00$$

where Asample is the absorbance of the mixture of phenolic sample, starch solution, enzyme and DNS colour reagent solution; A control is the absorbance of the mixture of buffer, starch solution and DNS colour reagent without enzyme; A test is the absorbance of the mixture of buffer (instead of the sample), starch solution, enzyme and DNS colour reagent; A blank is the absorbance of the mixture of phenolic sample, starch solution and DNS colour reagent without enzyme.

### α-glucosidase inhibition assay

The α-glucosidase activity was measured using a method of Oboh et al.^14^ and Tan et al.^25^ with a slight modification. Firstly, using 0.1 M phosphate buffer (pH 6.9) solution the reaction mixture was prepared as follows; 100 µl of appropriate dilutions of the extracts (0–10 mg/ml) or the standard polyphenols (0–350 µg/ml) and 100 µl of α-glucosidase (EC 3.2.1.20, 1U/mL) were mixed. Then the mixture was incubated at 37 °C for 10 min. Subsequently, 50 µl of 5 mM p-nitrophenyl-a-D-glucopyranoside, prepared with 0.1 M phosphate buffer (pH 6.9) solution was added to the mixture and incubation was repeated at the same conditions. Finally, the absorbances was read at 405 nm. The α-glucosidase inhibitory activity was expressed as a percentage of inhibition and calculated the same as the equation of α-amylase inhibition assay as previously mentioned.

### Statistical analysis

In this study, three replicates were conducted in each experiment. The results of enzyme inhibition were expressed as mean ± standard deviation (SD). The data were analysed using paired T-test and significance differences were accepted at *p* ≤ 0.05. The inhibitory concentration of 50% (IC_50_) was determined using linear regression analysis.

## Results and discussion

### Identification and quantification of polyphenols of Kluai Hin

To identify polyphenols of Kluai Hin (crude extracts, fresh and freeze-dried pulp and peel extracted with M:W (80:20) and A:W:A (50:49:1) by RP-HPLC-DAD. (−)-Gallocatechin was a major compound found in all fresh and freeze-dried pulp and peel. The highest content of (−)-gallocatechin found in freeze-dried pulp with M:W (80:20) extraction, followed by (+)-catechin and (−)-epicatechin, and the lowest content of gallic acid was found in fresh peel with M:W (80:20) extraction (24.14 ± 03 mg/100 g FW) (Table 1). (−)-Gallocatechin was eluted at retention time (Rt) 10.9 min at the maximum wavelength of 280 nm in comparison with retention times, diode array spectra of authentic standard. In addition, the authentic standard was spiked into samples for confirmation. Other compounds; i.e., gallic acid (Rt = 5.4, peak 1), (+)-catechin (Rt = 15.0, peak 4) and (−)-epicatechin (Rt = 17.4, peak 3) were also detected in Kluai Hin (Supplementary material 1 and 2).

**Table 1:** Content of polyphenols found in fresh and freeze-dried Kluai Hin (*Musa sapientum* Linn.) pulp and peel, extracted with methanol:water (M:W, 80:20) and acetone:water:acetic acid (A:W:A, 50:49:1).

Samples	Extraction solution	Content of polyphenols (mg/100 g FW)			
		Gallic acid	(−)-Gallocatechin	(+)-Catechin	(−)-Epicatechin
**Fresh**					
Pulp	M:W	ND	274.55 ± 00	ND	ND
	A:W:A	ND	139.25 ± 01	ND	ND
Peel	M:W	24.14 ± 03	766.55 ± 12	ND	74.66 ± 04
	A:W:A	ND	357.58 ± 04	459.33 ± 07	ND
**Freeze-dried**					
Pulp	M:W	ND	3204.69 ± 07	707.40 ± 15	ND
	A:W:A	ND	2108.31 ± 02	569.33 ± 09	ND
Peel	M:W	ND	1384.56 ± 21	411.60 ± 00	510.25 ± 16
	A:W:A	ND	1403.54 ± 00	629.66 ± 04	ND

Previous studies investigated the polyphenol content of different kinds of bananas, but their (−)-gallocatechin, (+)-catechin and (−)-epicatechin contents were detected in the pulp but were not quantified^3–7^. In another study, (−)-gallocatechin was identified in fresh Musa Cavendish banana peel (158 mg/100 g dry wt) and pulp (29.6 mg/100 g dry wt)^21^. In agreement with Huang et al.^3^ and Someya et al.^21^, the present study revealed that (−)-gallocatechin was the major compound analyzed by RP-HPLC-DAD with higher content. Other polyphenols in Thai banana, KluaiHom, were determined using isocratic HPLC-UV, and ferulic acid was found to be the main insoluble phenolic acid^26^. A local banana that grows in Malaysia (Pisang) was extracted with different solvents and the analysis method^27^. The extraction method and solvent types affected the composition and polyphenol content of bananas. For example, methanol worked better for flavonoids extraction^3^ while acetone was better for phenolic acids^5^. The current results indicated that Kluai Hin is a natural source of polyphenols, and its peel contains more phenolics than pulp. In addition, the type of polyphenols obtained from freeze-dried banana extracts was higher than that obtained from fresh banana extracts. It is possible due to freeze-dried process preserved the polyphenol content and antioxidant activity better than fresh^14^.

In this study, the total phenolic content of crude extracts, expressed as the GAE value (gallic acid equivalent, mg gallic acid/g fresh weight), was evaluated using the Folin-Ciocalteu method (Supplementary material 3). The phenolic content of fresh samples was higher than that of freeze-dried bananas. In contrast, Guine et al.^23^ found that freeze-dried bananas preserved the phenolic content better than a heat-treated banana. In this study, the preparation process of freeze-dried samples through 100 mesh sieves probably caused a significant loss of other polyphenols. Thus, the preparation step should be improved for future studies. Furthermore, peel samples contained more total phenolic content than the pulp of Kluai Hin, except in freeze-dried sample extraction with M:W (80:20) (*p* value < 0.05). The A:W:A (50:49:1) extraction solution presented a higher yield of total phenolic content than the extracted M:W (80:20). Alothman et al.^27^ revealed that acetone provided the highest yield of total flavonoid content in banana extracts due to the solubility of the polyphenols when compared with other solvent systems. Therefore, the A:W:A solvent was more effective in extracting Kluai Hin polyphenols.

However, some of the peaks were not identified in the RP-HPLC-DAD chromatogram, and unidentified peaks might be a source of polyphenols. After that, freeze-dried peel extracts, the richest polyphenolic profile, were further analysed by HPLC-ESI-QTOF-MS. As far as is known there have been no reports of detecting Kluai hin banana polyphenols using HPLC-ESI-QTOF-MS. Polyphenols were identified based on the exact mass of the compounds and fragmentation spectra. Both M:W and A:W:A peel extracts contain different types of polyphenols, phenolics and flavonoids and their derivatives. For phenolics, while chlorogenic acid and trans-p-coumaric acid 4-glucoside were detected in the M:W extract, only 3-O-caffeoyl-4-O-methylquinic acid was detected in the A:W:A extract. For flavonoids and derivatives, (−)-epicatechin 7-O-glucuronide, macrocarposide, 3,3′,4′,5,7-pentahydroxyflavan (4->8)-3,4′,5,7-tetrahydroxyflavan, vitexin 7-O-glucoside and 4′-O-methylneobavaisoflavone 7-O-(2″-p-coumaroylglucoside) were found in M:W extraction but saponarin and sophorapterocarpan A were found in A:W:A extracted peels. In addition, subclasses of hydroxycinnamic acids and flavonoids, flavanols, isoflavanone, flavone, and flavanone, were found in both the M:W and A:W:A extracts (Table 2).

**Table 2:** Polyphenols detected and characterized in Kluai Hin peel extracted with methanol:water (M:W, 80:20) and acetone:water:acetic acid (A:W:A, 50:49:1) by HPLC-ESI-QTOF-MS in negative ionization mode.

Extraction method	RT (min)	Tentative identification	Molecular formula	[M-H]-(m/z)	Major fragment ions (m/z)	Polyphenol class	
						Phenolic acids	Flavonoids and derivatives
M:W extraction	1.94	Quinic acid	C7 H12 O6	191.0561	191.0553	✓	
	10.89	Chlorogenic Acid	C16 H18 O9	353.0864	353.0851	✓	
	11.31	Glucocaffeic acid	C15 H18 O9	341.0873	161.0242	✓	
	12.45	(−)-Epicatechin 7-O-glucuronide	C21 H22 O12	465.1015	303.0496		✓
	12.60	Prunin 3″,6″-di-p-coumarate	C39 H34 O14	771.1936	771.1938		✓
	13.11	trans-p-Coumaric acid 4-glucoside	C15 H18 O8	325.0915	145.0290	✓	
	13.25	5Z-Caffeoylquinic acid	C16 H18 O9	353.0861	173.0449	✓	
	13.96	1-O-2′-Hydroxy-4′-methoxycinnamoyl-b-D-glucose	C16 H20 O9	355.1020	175.0401	✓	
	15.64	(±)-Catechin	C15 H14 O6	289.0712	289.0699		✓
	16.04	Macrocarposide	C21 H22 O11	449.1071	449.1070		✓
	16.45	3′-Glucosyl-2′,4′,6′-trihydroxyacetophenone	C14 H18 O9	329.0869	167.0344	✓	
	17.39	3,3′,4′,5,7-Pentahydroxyflavan(4->8)-3,4′,5,7-tetrahydroxyflavan	C30 H26 O11	561.1377	561.1373		✓
	19.95	Epiafzelechin (2R,3R)(−)	C15 H14 O5	273.0762	273.0760		✓
	28.60	Vitexin 7-O-glucoside	C27 H30 O15	593.1485	593.1482		✓
	28.68	Hovenitin I	C16 H14 O8	379.0658	185.0088		✓
	28.96	Perilloside E	C17 H22 O9	369.1175	369.1168	✓	
	28.98	4′-O-Methylneobavaisoflavone 7-O-(2″-p-coumaroylglucoside)	C36 H36 O11	643.2212	643.2210		✓
	29.07	Kuwanon Z	C34 H26 O10	639.1531	639.1522		✓
	29.10	Kuwanon K	C40 H36 O11	737.2254	737.2238		✓
	29.28	Eriodictyol	C15 H12 O6	287.0553	287.0555		✓
A:W:A extraction	1.94	Quinic acid	C7 H12 O6	191.0559	191.0553	✓	
	11.29	Glucocaffeic acid	C15 H18 O9	341.0864	161.0240	✓	
	12.53	Prunin 3″,6″-di-p-coumarate	C39 H34 O14	771.1926	771.1928		✓
	13.18	5Z-Caffeoylquinic acid	C16 H18 O9	353.0861	353.0851	✓	
	13.86	1-O-2′-Hydroxy-4′-methoxycinnamoyl-b-D-glucose	C16 H20 O9	355.1020	175.0397	✓	
	15.46	(±)-Catechin	C15 H14 O6	289.0706	289.0708		✓
	16.26	3′-Glucosyl-2′,4′,6′-trihydroxyacetophenone	C14 H18 O9	329.0863	167.0350	✓	
	19.78	Epiafzelechin (2R,3R)(−)	C15 H14 O5	273.0759	273.0758		✓
	28.60	Saponarin	C27 H30 O15	593.1483	593.1479		✓
	28.68	Hovenitin I	C16 H14 O8	379.0657	185.0086		✓
	28.91	3-O-Caffeoyl-4-O-methylquinic acid	C17 H20 O9	427.1241	429.1384	✓	
	28.96	Perilloside E	C17 H22 O9	369.1177	369.1177	✓	
	29.07	Kuwanon Z	C34 H26 O10	639.1535	639.1524		✓
	29.10	Kuwanon K	C40 H36 O11	737.2258	737.2259		✓
	29.28	Eriodictyol	C15 H12 O6	287.0552	287.0554		✓
	30.57	Sophorapterocarpan A	C20 H20 O4	323.1278	325.1829		✓

In another study, the polyphenol composition of plantain pulp and peel were analysed with HPLC-ESI-HR-MS and HPLC-DAD, and hydroxycinnamics was the major compound in pulp, and peel predominantly contained flavonol glycosides^5^. The difference in polyphenol composition might be due to genetic variation, species, area of growth or environmental conditions, harvest time and storage conditions^3,19,28,29^. Although the types and the content of polyphenols found in Kluai Hin banana were different from those in other banana types, due to its rich bioactive components, it is still beneficial to human health.

### Inhibition of α-amylase and α-glucosidase activity by crude extracts and comparison with acarbose in vitro

To compare A:W:A (50:49:1) and M:W (80:20) extraction, the effects of all the extracts from Kluai Hin were analyzed for their inhibitory activity on digestive enzymes. The α-amylase activity of the extracts was reduced in a concentration-dependent manner (Table 3).

**Table 3:** IC_50_ values (mg/ml) of crude extracts from pulp and peel (Methanol:Water (M:W) (80:20) and Acetone:Water:Acetic (A:W:A) (50:49:1)) Kluai Hin against α-amylase and α-glucosidase compared with acarbose.

Kluai Hin extracts	IC_50_ (mg/ml)	
	α-amylase	α-glucosidase
Fresh pulp + M:W (80:20)	5.59 ± 0.12	No inhibition
Fresh peel + M:W (80:20)	4.87 ± 0.19	1.98 ± 0.01
Fresh pulp + A:W:A (50:49:1)	6.29 ± 0.01	No inhibition
Fresh Peel + A:W:A (50:49:1)	2.66 ± 0.07	1.86 ± 0.02
Freeze-dried pulp + M:W (80:20)	7.49 ± 0.08	No inhibition
Freeze-dried peel + M:W (80:20)	6.90 ± 0.11	No inhibition
Freeze-dried pulp + A:W:A (50:49:1)	4.73 ± 0.01	No inhibition
Freeze-dried peel + A:W:A (50:49:1)	2.97 ± 0.00	1.99 ± 0.01
Acarbose (as a positive control)	0.25 ± 0.01	0.14 ± 0.01

The IC50 values of the fresh and freeze-dried peel of banana extracts were 2.66 ± 0.07 mg/ml and 2.97 ± 0.00 mg/ml, respectively. There were approximately 10 times higher than the positive control (0.25 mg/ml). Another study reported that the inhibitory effect of Kluai Hom Thong (Musa AAA group), Kluai Nam Wa (Musa ABB group) and Kluai Khai (Musa AA group) bananas on α-amylase ranged between 7.22 ± 0.41 mg/ml and 15.5 ± 0.02 mg/ml, and the purified α-amylase inhibitor had similarities to the plant chitinase family^30^. The findings of our study revealed that Kluai Hin shows a stronger inhibitor of α-amylase activity than other types of Thai bananas.

In this study, Kluai HIn peel inhibited α-glucosidase activity; however, pulp did not exhibit any inhibitory activity of α-glucosidase. It ranged from 1.86 ± 0.02 mg/ml to 1.99 ± 0.01 mg/ml or 13 times higher than acarbose (0.14 ± 0.01 mg/ml). Pulp and peel extracts showed lower inhibition of α-amylase and α-glucosidase than the therapeutic inhibitor acarbose. Thus, Kluai Hin can be used as a source of functional food for the prevention of diabetes but not as a therapeutic drug to treat diabetic patients. The result from this study may encourage health-conscious consumers to consume more Kluai Hin bananas.

Furthermore, Kluai Hin consumption probably provides an alternative health benefit similar to another type of Thai banana called Kluai Khai. For example, Leerach et al.^31^ reported that the intake of Kluai Khai prevented UVB-induced skin damage in mice. Hence, it possibly provides a low risk of premature skin ageing and carcinomas. In addition, the resistant starch content, the in vitro starch digestibility and physico-chemical properties of flour and starch from Thai bananas, including Kluai Hin, were analysed, and it was stated that four starches from these bananas are possibly suitable for creating healthy food products^32^. Not only Kluai Hin pulp but an active compound from its peel will be used as natural antioxidants in food products^33–35^.

Furthermore, several studies reported that there are only polyphenols but not the other secondary metabolites (phytosterols, terpenoids) are explored in a different part of banana (such as flower, pseudostem and peel)^8,17^. These compounds can inhibit enzymes responsible for the α-glucosidase and insulin levels. Additionally, some phytosterols can enhance the glycolytic activities of enzymes in diabetic rats, resulting in hepatic glucose utilization^36^. Thus, Kluai Hin peel probably contains similar types of phytosterols and is capable to control blood glucose levels with a similar mechanism of action. Therefore, the value of local banana, Kluai Hin, will be added and higher utilized for food development instead of discarded as waste.

### Inhibition of α-amylase and α-glucosidase activity by authentic standards; (+)-catechin, (−)-epicatechin, (−)-gallocatechin and gallic acid compared with acarbose in vitro

Huang and colleagues^3^ reported that (−)-gallocatechin, (+)-catechin and (−)-epicatechin were detected in banana pulp, but no study of α-amylase and α-glucosidase inhibitory effects was performed. Therefore, the effects of (−)-gallocatechin, (+)-catechin, (−)-epicatechin and gallic acid on α-amylase and α-glucosidase inhibitory activities were studied and are presented in Supplementary material 4.

The results revealed that all four polyphenols had no α-amylase inhibitory effect but activation instead. The concentration of authentic standards ranged between 0 and 350 µg/ml. While the positive control (acarbose) inhibited α-amylase activity by approximately 65%, (−)-gallocatechin, (+)-catechin, and (−)-epicatechin did not inhibit α-amylase activity, possibly due to a lack of standard concentrations. Other factors, such as enzyme concentration, substrate type, incubation time, temperature, and pH, need to be considered. Additionally, the reducing potential of polyphenols may interfere with the development of the colour of 3,5 di-nitrosalicylic acid in the reaction mixture^12^. Our study showed that the inhibitory effect of all four polyphenols on α-glucosidase was dose-dependent. The IC_50_ values of (+)-catechin, (−)-epicatechin, (−)-gallocatechin and acarbose ranged from 100.40 ± 14.33 µg/ml to 588.08 ± 14.21 µg/ml. The inhibitory activities of (+)-catechin and (−)-gallocatechin were not different and higher than that of acarbose (Supplementary material 4B). In addition, the inhibitory activity was lowest in (−)-epicatechin and no inhibition in gallic. (+)-Catechin had an inhibitory effect on α-glucosidase. The inhibitory activity of (+)-catechin at 50% was 100.40 ± 14.33 µg/ml, approximately 2 times higher than Tadera et al. (45% at a concentration of 200 µM, 58.05 µg/ml)^37^. In this study, the gallocatechin inhibitory activity on α-glucosidase was investigated and found 143.34 ± 7.40 µg/ml at 50%. (−)-Epicatechin inhibited α-glucosidase at 50% (588.08 ± 14.21 µg/ml). Tadera et al.^37^ found that the α-glucosidase inhibition of (−)-epicatechin was 24% at concentrations greater than 200 µM (approximately 58 µg/ml). The inhibition effect of (−)-epicatechin of this result was 5 times higher. Wang et al.^38^ found that the inhibitory effect of gallic acid was 8.05 ± 0.7% at 40 µg/ml, while this study showed no inhibitory effect of gallic acid. As mentioned before, this could be caused by factors such as pH, temperature, the volume of enzyme or substrate. Acarbose had an inhibitory effect on α-glucosidase. The inhibitory activity of acarbose at 50% was 177.18 ± 9.24 µg/ml.

Although authentic standards in this study showed that they were not effective in treating diabetes at the same amount as acarbose, these standards may affect indirect mechanisms of diabetes or other mechanisms^13,16^. For example, gallocatechin exhibited anti-inflammatory activity by decreasing NOS2 in LPS + IFN-γ-treated RAW 264.7 cells^39^. Thus, the pathogenesis of several disorders, including diabetes, could not be caused by inflammation.

According to the HPLC-ESI-QTOF-MS results in Table 2, the predominant active polyphenols explored in Kluai Hin peel were 3-O-caffeoyl-4-O-methylquinic acid (phenolic) saponarin (flavone glycoside) and sophorapterocarpan A (flavonoid) of A:W:A extracts. Therefore, these compounds may reveal specific inhibitory effects of α-amylase and α-glucosidase activity.

## Conclusions

To our knowledge, a polyphenol profile was identified for the first time in Kluai Hin by RP-HPLC-DAD and HPLC-ESI-QTOF-MS. The most abundant polyphenol, (−)-gallocatechin, was found in the crude extracts of Kluai Hin peel and pulp. Furthermore, different types of phenolics, flavonoids and derivatives of peel extracts were also detected by HPLC-ESI-QTOF-MS in acetone:water: acetic acid (50:49:1) or methanol: water (80:20). The predominant active polyphenols explored in the Kluai Hin peel were 3-O-caffeoyl-4-O-methylquinic acid saponarin and sophorapterocarpan A of A:W:A extracts.

Kluai Hin extracts revealed inhibition of the key enzymes of carbohydrate digestion, α-amylase and α-glucosidase, but lower levels than the therapeutic drug acarbose. On the other hand, the polyphenol standards explored in Kluai Hin (−)-gallocatechin, (+)-catechin, (−)-epicatechin and gallic acid activated α-amylase activity; however, (−)-gallocatechin, (+)-catechin, and (−)-epicatechin standards dose-dependently inhibited α-glucosidase inhibition. 3-O-Caffeoyl-4-O-methylquinic acid, saponarin and sophorapterocarpan A of A:W:A banana extracts from HPLC-ESI-QTOF-MS may inhibit these digestive enzymes instead. The combination of phenolic compounds in the extracts may inhibit both α-amylase and α-glucosidase activity; therefore, when the standards were analysed alone, they activated α-amylase activity and inhibited α-glucosidase activity.

Kluai Hin pulp, especially peel, is a natural source of polyphenols. These polyphenols will be able to decrease blood glucose levels by inhibiting α-amylase and α-glucosidase activity after Kluai Hin pulp intake. Therefore, consumption of Kluai Hin banana may decrease the risk of diabetes of health-conscious consumers or  improve the quality of life of prediabetic /diabetic patients.

## Supplementary Information

Supplementary Information.

## Abbreviations

AWA: Acetone:water:acetic
DAD: Diode array detector
ESI: Electrospray ionization source
FW: Fresh weight
GAE: Gallic acid equivalent
HPLC: High performance liquid chromatography
MS: Mass spectrometer
MW: Methanol:water
QTOF: Quadrupole-time of flight
RP: Reverse-phase
