# Supplementary material for: Kluai Hin (Musa sapientum Linn.) peel as a source of functional polyphenols identified by HPLC-ESI-QTOF-MS and its potential antidiabetic function
Source: Sci Rep. 2022 Mar 9;12:4145. doi: 10.1038/s41598-022-08008-3 (PMC8907229; doi:10.1038/s41598-022-08008-3)
Supplement: Supplementary file 1 — Supplementary Information. [file 41598_2022_8008_MOESM1_ESM.docx]

**3**

**2**

**1**

**A.**

**3**

**2**

**B.**

Supplementary material 1 Chromatograms of of polyphenols of fresh Kluai Hin peel, extracting with A) Mathanol:Water (80:20) and B) Acetone:Water:Acetic acid (50:49:1). Peaks 1) Gallic acid, 2) (-)-Gallocatechin and 3) (-)-Epicatechin.

**4**

**2**

**3**

**4**

**2**

**A.**

**B.**

Supplementary material 2 Chromatograms of of polyphenols of freeze-dried Kluai Hin peel, extracting with A) Mathanol:Water (80:20) and B) Acetone:Water:Acetic acid (50:49:1). Peaks 2) (-)-Gallocatechin 3) (-)-Epicatechin and 4) (+)-Catechin.

Supplementary material 3 Total phenolic content of Kluai Hin (Musa sapientum Linn.) pulp and peel (mg GAE/100g Fresh weight) extracted with methanol: water (80:20) and acetone: water: acetic (50:49:1)

| **Samples** | **Total phenolic content (mgGAE/100g Fresh weight)** | | | |
| --- | --- | --- | --- | --- |
|  | **Methanol:Water**  **(80:20)** | | **Acetone:Water:Acetic (50:49:1)** | |
|  | **Pulp** | **Peel** | **Pulp** | **Peel** |
| Fresh | 533.40±4.80 | 793.53±14.13 | 637.17±17.66 | 811.56±7.94 |
| Freeze-dried | 582.97±6.95 | 394.96±9.07 | 527.10±6.20 | 565.03±11.37 |

**A.**

**B.**

Supplementary material 4 Inhibitory effects of authentic standards of (-)-gallocatechin, (+)-catechin, (-)-epicatechin and acarbose on α-amylase (A) and α-glucosidase (B)
